# Disease characteristics and causes of early and late death in a group of Croatian patients with systemic lupus erythematosus deceased over a 10-year period

**DOI:** 10.3325/cmj.2018.59.3

**Published:** 2018-02

**Authors:** Ivan Padjen, Mislav Cerovec, Marijan Erceg, Miroslav Mayer, Ranko Stevanović, Branimir Anić

**Affiliations:** 1 Division of Clinical Immunology and Rheumatology, Department of Internal Medicine, University Hospital Center Zagreb and University of Zagreb, School of Medicine, Zagreb, Croatia; 2 Croatian Institute of Public Health, Zagreb, Croatia

## Abstract

**Aim:**

To assess the causes of early death (ED) and late death (LD) in patients with systemic lupus erythematosus (SLE) and determine the features of deceased SLE patients followed-up in a single Croatian tertiary hospital center, because little if any data on causes of death (CODs) in SLE patients are available for Croatia.

**Method:**

We identified SLE patients regularly followed-up at the Division of Clinical Immunology and Rheumatology, University Hospital Center Zagreb, who died from 2002 to 2011. Death was ascertained by matching our institutional records with the Croatian National Death Database. Patients were grouped according to their disease duration to ED and LD and compared by demographic characteristics, classification criteria, organ damage, and CODs.

**Results:**

We identified 90 patients (68 women), who died at the age of 58 ± 15 years. The most frequent COD category was cardiovascular diseases (40%), followed by infections (33%), active SLE (29%), and malignancies (17%). No significant difference was found between the frequencies of causes of ED and LD, except for stroke, which caused only LD≥10 years after the diagnosis. SLE was reported in death certificates of only 41 of 90 patients.

**Conclusion:**

Although stroke occurred both in the early and late disease course, it was primarily associated with LD. Given the low proportion of SLE recorded in death certificates of deceased SLE patients, matching of institutional and vital statistics records may be required to assess the true impact of SLE on mortality.

The disease course and causes of death in patients with systemic lupus erythematosus (SLE) are characterized by a complex interplay of the systemic disease itself and a number of comorbidities (1). In the 1970s, Urowitz et al (2) described a bimodal pattern of mortality in SLE patients, with active disease and infections as leading causes of early death and cardiovascular diseases as the major cause of late death. A similar pattern was observed in their prospective study of 51 patients one decade later (3).

However, the bimodal pattern has not been unequivocally recognized in the new millennium. It was confirmed in a large multicenter Chinese study (4), but not in the European multicenter study of causes of death (5). Nonetheless, concepts of early and late death remain generally accepted. Five years after SLE diagnosis and/or meeting the classification criteria, primarily those of the American College of Rheumatology (ACR), have been a provisional time point accepted as a cut-off between early and late death in previous studies (5-9).

Over the last four decades, a tremendous improvement in outcomes of SLE has been observed both in Europe and worldwide. Increase in a 5-year survival from 50% to over 90% and the achievement of 10-year survival exceeding 90% redirected the focus of interest toward the features of long-term disease, including causes of late death (5,9-11).

Most data on SLE outcomes in Europe are derived from studies conducted in established cohorts of SLE patients in highly developed countries with health care systems able to support dedicated lupus centers. However, SLE is a disease with pronounced phenotypic differences between geographically, ethnically, and socio-economically different populations. Given a relatively loose SLE definition, reliance on clinicians’ evaluation, no diagnostic criteria, and a set of classification criteria that include only the most specific features of SLE, these differences are even more pronounced than those related to some other disorders, such as arterial hypertension, coronary heart disease, or asthma. Although our Center participated in the aforementioned multicenter European study on causes of death (5), little if any data are available on the outcomes of SLE in Croatia and neighboring central and southeastern European countries and causes of death and features of deceased SLE patients from Croatia have never been specifically analyzed.

Our aim was to assess causes of death and disease features in SLE patients followed-up regularly in our institution who died over a 10-year period, with an emphasis on differences between early and late death. Our hypothesis was that active disease and infections were more frequent causes of early death, whereas cardiovascular diseases and malignant tumors more frequently caused late death.

## PATIENTS AND METHODS

### Patients

**Figure 1:**
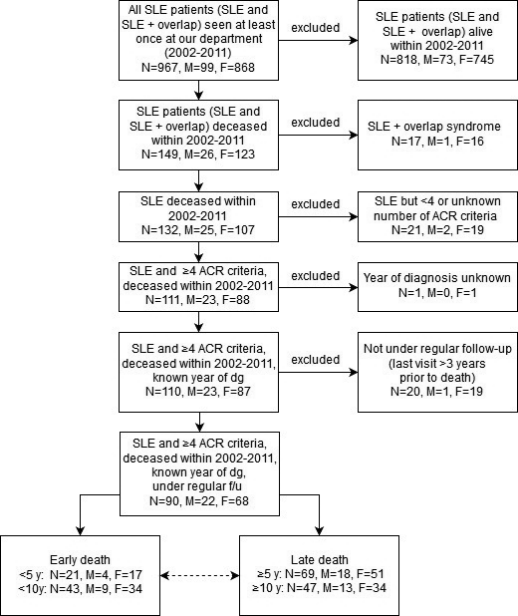
Flow diagram of selection of patients with systemic lupus erythematosus (SLE) from 2002 to 2011. Dotted two-way arrow indicates comparison of early and late death. Meeting ≥4 American College of Rheumatology (ACR) criteria was used as a cut-off for classification of SLE patients according to the criteria definition. Abbreviations: N – number of patients; M – number of males; F – number of females; dg – diagnosis; f/u – follow-up; y – years; SLE + overlap – patients with a diagnosis of SLE and another systemic autoimmune disease.

This retrospective observational study included patients with SLE who visited the Division of Clinical Immunology and Rheumatology, University Hospital Center Zagreb, at least once between January 1, 2002 and December 31, 2011. The Division serves as the leading academic center dedicated to connective tissue diseases and SLE in Croatia. Of all SLE patients who visited the Division, we identified regularly followed-up patients who died in the study period and who were Croatian residents at the time of death, fulfilled ≥4 ACR criteria (6,7), and had a known year of diagnosis (Figure 1). A patient was considered to be regularly followed-up if the last visit to our institution had been within three years before death (12). We excluded patients with an overlap of SLE and another systemic autoimmune disease.

The patients were included in the early death (ED) group if death occurred in the early disease course and in the late death (LD) group if death occurred in the late disease course. Disease duration was defined as time from diagnosis to death. Two cut-off points between early and late death were used, ie, five and ten years after diagnosis, as proposed by Nossent et al (5).

### Data sources and collection

Data were extracted from medical charts stored at the Division since 1978 and from the Division’s computer database of SLE patients, containing demographic data and main disease features. The deceased patients were identified by matching the Division’s database with the National Death Database administered by the Croatian Institute of Public Health. The National Death Database contains data on a single cause of death for each patient who died after 2002. Causes of death were identified from medical charts and from the National Death Database. They were also obtained from primary care physicians and by direct contact with family members, when available.

Distance between the patients’ residence (home address) and our institution (in kilometers) were obtained by determining the shortest route provided by the internet-based service Google Maps (Google LLC, Mountain View, California, USA).

For each patient, we collected demographic data, number of ACR criteria met at diagnosis and time of death (6,7), damage according to the Systemic Lupus International Collaborating Clinics (SLICC)/ACR damage index (SDI) one year after diagnosis as an indicator of early damage and cumulatively at time of death (13), causes of death, and medications.

Data on ACR criteria met at diagnosis were missing for 17 of 90 patients (supplementary Material 1(supplementary material 1)). Causes of death were grouped into categories most frequently mentioned in the literature as follows: active lupus, infections, cardiovascular diseases, and malignant tumors (5,8,9,14,15). Death was attributed to active lupus provided there was clinical or *post mortem* evidence of active disease (16). More than one cause of death was possible in a single patient; however, the cause of death was unknown in 8 of 90 patients. Medications included glucocorticoids and immunosuppressive agents such as oral cyclophosphamide, intravenous cyclophosphamide pulses, cyclosporine A, azathioprine, and mycophenolate mofetil. Data on the treatment with warfarin and acetylsalicylic acid were collected for patients who died from stroke.

### Statistical analysis

Continuous data were presented descriptively as mean and standard deviation (SD) or median and interquartile range (IQR). Categorical variables were presented as proportions or frequencies.

Data were compared between the ED and LD groups. As an exception, proportions or frequencies of patients treated with glucocorticoids and immunosuppressive agents were compared between patients who died due to an infectious cause and patients who died due to a non-infectious cause.

The Kolmogorov-Smirnov test was used to test for normality of distribution. Normally distributed continuous variables were compared using the Student *t*-test, whereas the Mann-Whitney U-test was used in other cases. Categorical variables were compared using the χ^2^ test and Fisher exact test. As an exception, comparison of damage accrual during the first year after diagnosis and the annual accrual of damage during further follow-up (both variables non-normally distributed) was performed using the Wilcoxon signed rank test, suitable for paired observations.

*P* values less than 0.05 were considered statistically significant except in cases of adjustment for multiple comparisons, where the cut-off for statistical significance was decreased according to the Holm-Bonferroni method (17).

Correlation between the disease duration defined as time from SLE diagnosis to death and distance between patient place of residence and our institution was presented using the Spearman’s rank correlation coefficient (due to a non-normal distribution of the correlated data) with 95% confidence interval (CI).

Statistical analyses were performed using Statistica ver. 12 (Dell, Round Rock, Texas, USA; license issued to the School of Medicine, University of Zagreb), except for the distribution of mortality and the existence of its (bi)modal pattern, which was analyzed using the Joinpoint Regression Program version 4.2.0.2 (National Cancer Institute, Bethesda, MD, USA) (18).

## RESULTS

From 2002 to 2011, a total of 967 SLE patients visited the Division. Of them, 149 died during the 10-year period. The study criteria were met by 90 patients, of whom 21 died within five and 43 within 10 years after diagnosis (Figure 1).

### Demographic characteristics

**Figure 2:**
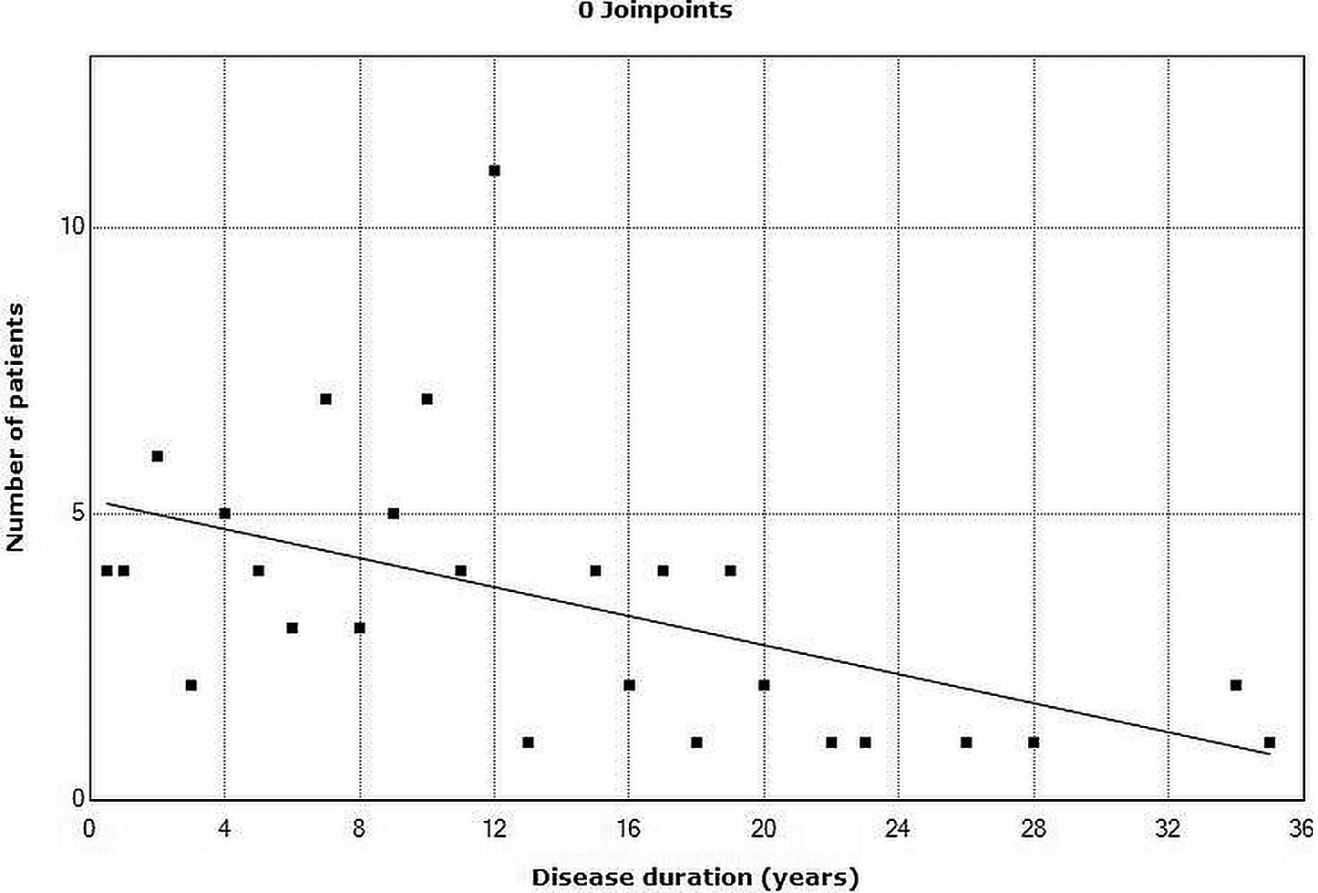
Distribution of mortality in 90 patients with systemic lupus erythematosus (SLE). A (bi)modal pattern was not confirmed. Number of patients refers to the patients deceased at a time point of X years after SLE diagnosis.

There were three times more women than men among the SLE patients included in our study (Table 1). The mean age at death for all patients was ≤60 years, with no difference between the ED and LD groups. Patients in the ED group were diagnosed at an older age than patients in the LD group. The mean disease duration was 10.6 ± 7.7 years. Joinpoint regression revealed zero joinpoints, yet only a slightly declining trend in the number of deaths that occurred as a function of time elapsed from diagnosis (Figure 2). In other words, a (bi)modal pattern was not observed.

**Table 1:** Characteristics of 90 patients with systemic lupus erythematosus who died between 2002 and 2011*

Characteristics	Patients grouped by disease duration						
	total	early death (<5 years)	late death (≥5 years)	*P*	early death (<10 years)	late death (≥10 years)	*P*
Women-to-men ratio	3.09	4.25	2.83	0.511	3.78	2.62	0.458
Age at diagnosis (years; mean±SD)	47.5 ± 16.9	56.4 ± 15.0	44.8 ± 16.6	0.005	55.7 ± 15.2	40.1 ± 15.0	<0.001
Age at death (years; mean±SD)	58.1 ± 14.8	58.4 ± 14.7	58.0 ± 14.9	0.914	60.3 ± 15.2	56.2 ± 14.3	0.187
Home-to-clinic distance (km; median, IQR)	46.8 (9.9-104.5)	49.8 (15.0-103.0)	41.9 (9.1-107.0)	0.501	49.8 (9.4-106.0)	41.5 (10.1-104.0)	0.796
Number of met ACR criteria (median, IQR)							
at diagnosis	4 (3-5)	4 (4-5)	4 (3-5)	0.038	4 (4-5)	3.5 (3-4)	0.009
at death	6 (5-7)	5 (4-6)	6 (5-7)	0.018	5 (4-6)	6 (5-7)	0.046
SDI (median, IQR)							
at 1 year after diagnosis	1 (0-2)	2 (1-3)	0 (0-1)	0.005	1 (0-3)	0 (0-1)	<0.001
at death	5 (3-7)	3 (1-4)	5 (3-8)	<0.001	3 (1-5)	7 (4-9)	<0.001

*Abbreviations: SD – standard deviation, IQR – interquartile range, ACR – American College of Rheumatology, SDI – Systemic Lupus Erythematosus International Collaborative Clinics/American College of Rheumatology (SLICC/ACR) Damage Index.

The median distance between patient place of residence (home address) and our clinic was 46.8 km (IQR 9.9-104.5 km). Among the 90 deceased patients, there was no correlation between the home-to-clinic distance and disease duration (Spearman’s rank correlation coefficient -0.1223, 95% CI -0.327 to 0.093).

### Disease features according to ACR criteria

In comparison with patients in the ED group, those in the LD group met a higher number of criteria at the time of death. Conversely, the number of criteria fulfilled at diagnosis was higher in the ED group (Table 1).

**Figure 3:**
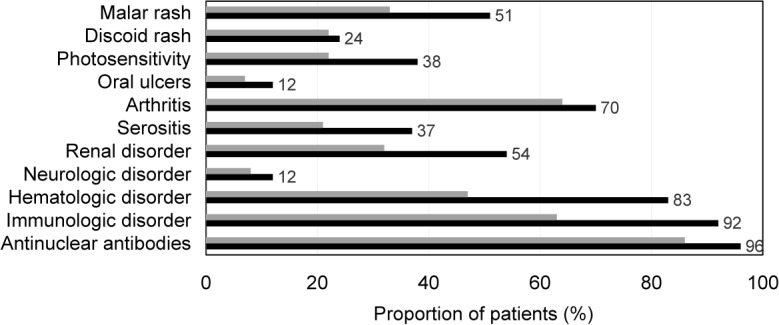
Distribution of the American College of Rheumatology classification criteria in 90 patients with systemic lupus erythematosus at the time of diagnosis (gray bars) and time of death (black bars).

The most frequent classification criteria observed among deceased patients both at diagnosis and cumulatively were related to the laboratory features of SLE and included antinuclear antibodies (ANA) and immunologic and hematologic disorders (Figure 3). Immunologic disorder was mostly driven by the presence of anti-double stranded DNA (anti-dsDNA) antibodies (79%), whereas hematologic disorder (83% cumulatively) was primarily driven by lymphopenia (64%) and leukopenia (49%). The most frequent clinical feature was arthritis (70% cumulatively). Despite the differences in the total number of criteria, no difference was observed between the ED and LD groups in the frequency of individual criteria (supplementary material 1(supplementary material 1)).

### Damage according to the SDI

Patients in the LD group accrued more damage over the course of disease than patients in the ED group, although their damage was lower one year after diagnosis (Table 1). Only 5 of 90 patients had no damage accrual over the entire disease course. Four of these patients died within five years, whereas the fifth patient died nine years after diagnosis. One year after diagnosis, damage accrual (early damage) was observed in 14 of 17 patients deceased within five years, 15 of 22 deceased 5-10 years after diagnosis, and only 19 of 47 patients deceased after ≥10 years. In the group of patients who died within five years after diagnosis, the denominator is 17 rather than 21, because four patients died in less than a year after diagnosis.

**Figure 4:**
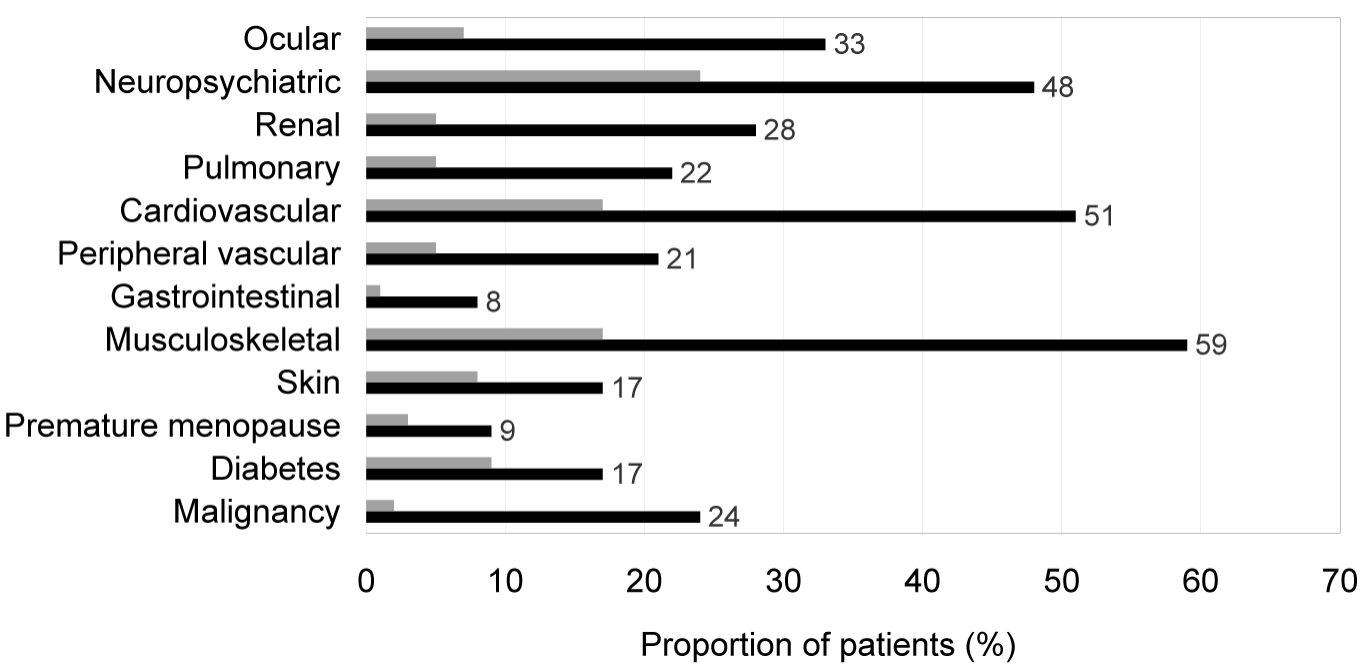
Distribution of damage according to the Systemic Lupus International Collaborating Clinics/American College of Rheumatology Damage Index (SDI) in 90 patients with systemic lupus erythematosus 1 year after diagnosis (gray bars) and at time of death (black bars).

The most frequently observed components of the SDI, both at death and one year after diagnosis, were musculoskeletal, cardiovascular, and neuropsychiatric damage (Figure 4). Comparison of individual components of the SDI revealed a higher proportion of cumulative musculoskeletal damage in patients deceased ≥5 years after diagnosis (48 of 69 patients) compared to those deceased <5 years after diagnosis (5 of 21 patients; *P* < 0.001). It also revealed a higher proportion of cardiovascular damage one year after diagnosis in patients deceased <10 years after diagnosis compared to patients deceased thereafter (13 of 39 vs 2 of 47 patients; *P* < 0.001) (supplementary material 2(supplementary material 2)). Majority of the (sub)components of the SDI were observed in at least one patient within a year after diagnosis (supplementary material 3(supplementary material 3)).

In all patients, damage accrued during the first year after diagnosis of SLE (median 1, IQR 0-2) exceeded annual damage accrual over the following disease course (median 0.33, IQR 0.09-0.51) (*P* < 0.001, Wilcoxon sum of signed ranks equaling 1685).

### Causes of death

No difference was observed in the frequencies of causes of death between the ED and LD groups irrespective of whether the 5- or 10-year cut-off was used (Table 2). The exception was stroke, which caused death only ≥10 years after diagnosis, even though patients in the ED group also developed stroke as a component of damage during the course of their disease.

**Table 2:** Distribution of causes of death in patients with systemic lupus erythematosus according to categories and comparison of their frequencies between early and late death

Cause of death*	No. (%) of patients						
	total (N = 90)	early death (<5 years) (n = 21)	late death (≥5 years) (n = 69)	*P*	early death (<10 years) (n = 43)	late death (≥10 years) (n = 47)	*P*
Active lupus	26 (29.0)	8	18	0.288	11	15	0.508
Infection	30 (33.0)	9	21	0.290	14	16	0.881
sepsis	20 (22.9)	6	14	0.549	9	11	0.778
pneumonia	18 (20.0)	5	13	0.756	8	10	0.797
urinary tract infection	8 (9.0)	3	5	0.329	5	3	0.472
Cardiovascular disease	36 (40.0)	6	30	0.222	16	20	0.605
ischemic cardiomyopathy	7 (13.0)	1	11	0.281	6	6	0.869
myocardial infarction	5 (6.0)	1	4	1.000	1	4	0.363
stroke	8 (9.0)	0	8	0.190	0	8	0.006
pulmonary embolism	3 (3.0)	1	2	0.554	1	2	1.000
Malignant tumor	15 (17.0)	1	14	0.177	4	11	0.073
Other	10 (11.0)	4	6	NT^§^	6	4	NT^†^
Unknown	8 (8.0)	1	7	NT^§^	2	6	NT^†^

*More than one cause possible in a single patient.

†Not tested.

Despite no difference in the observed frequencies, a difference in their sequence could be seen between patients deceased within and after five years following diagnosis. While infection and active disease were the most frequent causes of death in patients deceased within five years, cardiovascular diseases were the leading cause in patients deceased after five years following diagnosis.

Among 26 patients that succumbed to active lupus, 17 patients had multiorgan activity (≥2 organs affected), six had active renal disease, two had active neurolupus, and a single patient had vasculitis. There was a notable overlap between disease activity and infection, with 14 of 26 patients also having infection reported as a cause of death. Furthermore, 4 of 8 patients deceased due to stroke also died from active lupus.

All 30 patients who deceased due to infections were treated with glucocorticoids during their disease course, whereas 22 of them also received immunosuppressive agents (11 patients received oral cyclophosphamide, 7 received cyclophosphamide intravenous pulses, 11 received azathioprine, 3 received cyclosporine A, and 1 patient received mycophenolate mofetil). Similar proportions, but without statistically significant differences in comparison with the previous 30 patients, were observed in the remaining 60 patients who died due to non-infectious causes. Of these 60 patients, 59 were treated with glucocorticoids (*P* = 0.477 for comparison with the infectious disease group), and 33 of 60 received immunosuppressant drugs (*P* = 0.093) including oral cyclophosphamide in 17 patients (*P* = 0.421), cyclophosphamide intravenous pulses in 8 patients (*P* = 0.230), azathioprine in 25 patients (*P* = 0.648), cyclosporine A in 3 patients (*P* = 0.396), and mycophenolate mofetil in 1 patient (*P* = 1.000). Of 36 patients deceased due to cardiovascular disease, 15 succumbed to a thrombotic or thromboembolic event, such as stroke, myocardial infarction, and pulmonary embolism (Table 2). One patient had both stroke and myocardial infarction listed as causes of death. Among these 36 patients, six had secondary antiphospholipid syndrome (APS), 17 patients were positive for anticardiolipin antibodies (ACLA), and six had a positive lupus anticoagulant (LAC). LAC positivity was observed in five patients also having positive ACLA. Although antibodies to beta-2 glycoprotein I (beta-2-GPI) were found in two patients also positive for LAC and ACLA, detection of beta-2-GPI antibodies was not routinely available at our institution until 2010. Among 15 patients deceased due to a thrombotic or thromboembolic event, four were diagnosed with secondary APS, six were positive for ACLA, two for LAC, and one for antibodies to beta-2-GPI. Of eight patients who died from stroke, four had been receiving acetylsalicylic acid (ASA) before stroke, 7 received ASA after the onset of stroke, and 2 were receiving warfarin. Warfarin had been prescribed before the onset of stroke due to episodes of deep venous thrombosis in conjunction with positive ACLA. In addition to thrombotic or thromboembolic events and ischemic cardiomyopathy, other cardiovascular causes of death included heart failure due to mitral valve insufficiency (2 patients), complete atrioventricular block (1 patient), ventricular tachycardia (1 patient), aortal dissection with cardiac tamponade (1 patient), hypertensive cardiomyopathy with atrial fibrillation (1 patient), sudden cardiac death (2 patients), and heart failure with cause not specified (6 patients).

Both solid and hematological malignancies were identified as causes of death. All were diagnosed after a median of 10 years (IQR 7-12 years) after SLE diagnosis. A total of 15 patients (13 women) died due to a malignant tumor. Lung, anal, and pancreatic carcinomas caused death in two patients each, whereas mesenterial non-Hodgkin lymphoma, myeloproliferative syndrome, prostatic, breast, bladder, renal, ovarian, cervical, and colorectal carcinoma caused death in a single patient each. In this group of patients SLE was diagnosed at a mean age of 46 ± 14 years. Baseline data on ACR criteria met at diagnosis of SLE were available for 13 of these 15 patients and included malar rash (7 patients), discoid rash (3 patients), photosensitivity (7 patients), and arthritis (5 patients). Antinuclear antibodies were positive in 10, antibodies to double-stranded DNA in 6, and ACLA in 2 patients. Leukopenia was detected in 3 of the 13 patients as the only hematological feature. Conversely, renal (proteinuria and urinary cellular casts) and neuropsychiatric disorder (psychosis) were observed each in a single patient, while serositis was not observed at diagnosis of SLE in any of the 13 patients.

Autopsy was performed in 17 of 90 patients. SLE was reported in the death certificate of only 41 of 90 patients. Of 90 deceased patients, 63 died in the hospital setting, 23 at home, and 4 in a retirement home.

## DISCUSSION

No difference was found in the frequencies of causes of death between the ED and LD groups. Therefore, our hypothesis was not confirmed. The only exception was stroke that caused exclusively late death. Because stroke was also observed in patients who died earlier, although not as a cause of death, stroke occurring ≥10 years after diagnosis may be associated with increased mortality. Despite the prevailing perception of stroke as a consequence of atherosclerosis (19), disease activity may also play a role in its development (20).

The difference in the sequence of causes of death between patients deceased within and after five years is consistent with the perception that infection and active SLE are causes of early death and cardiovascular diseases are causes of late death (2,21). However, our analysis did not confirm the bimodal pattern of mortality, but corresponded with an almost linear distribution of mortality as found in the multicenter European study by Nossent et al (5). The “flattening” of the bimodal curve may be attributed not only to better control of the disease, but also to increased recognition of patients with less severe SLE. A further similarity to that study is the lack of difference in the frequencies of causes of early and late death, except for active SLE that was a more frequent cause of early death in the study by Nossent et al (5). Similar to our study, stroke caused only late death in the Korean study by Kang et al (8) and the British study by Moss et al (9). Except for stroke, Korean patients had no other atherosclerosis-related causes (8), which is in contrast to European populations, but consistent with other studies of SLE in Asian patients (16,22). Pulmonary hypertension caused death in a noticeable proportion of Korean patients (8) as opposed to European patients (5,9,14,23). Malignant tumors were more frequently observed in our study than in two European multicenter studies (5,14), and especially the Korean study where only one patient succumbed to malignant disease (8). The proportion of malignancies in our study was similar to proportions seen in two cohorts in the United Kingdom (9,23).

Although the proportion of unknown causes was up to 10% in our study and two previous studies (5,9), it exceeded 20% in the Euro-lupus project (14). The finding by Moss et al (9) that all patients with an unknown cause of death died >5 years after diagnosis may signal a contribution of disease duration and loss to follow-up to losing data. Furthermore, the rather low percentage of patients deceased outside the hospital in the study by Nossent et al (5) raises concerns about underrecognition of out-of-hospital death. Due to the relatively high extent of loss to follow-up in SLE patients, a linkage with sources of data other than hospital charts and death certificates of patients deceased in study hospitals seems to be required (8,24,25).

Multiple cause of death analysis (MCDA) may be a useful method to assess the mutual impact of different causes of death (26). Hitherto, two studies analyzed adult lupus mortality using this methodology (27,28). However, MCDA does not take into account lupus patients not having SLE reported in their death certificates. Underreporting of SLE was observed even in two prospective lupus cohorts from the United States, where SLE was not listed in 40% of death certificates (29). Underreporting was even more pronounced in our study.

Similar to the study by Nossent et al. (5), damage at time of death in our study was higher in the LD than in ED group. In the aforementioned study, mean damage at late death was 7.2, while it was 4 in patients deceased earlier (5). Both figures are higher than in our study, despite our patients being >10 years older at diagnosis. Possible explanations of this difference are higher frequencies of criteria associated with severe disease in the study by Nossent et al. (5) and lower percentage of in-hospital deaths in our study, potentially suggestive of less severe disease. Another possible explanation is that the starting point of follow-up in our study was defined as diagnosis of SLE, as it was in the study by Kang et al (8), whereas Nossent et al (5) used the time of fulfilling ≥4 criteria as the starting point. Although the disclosed disease duration in our and two other studies was around 10 years (5,8), the actual time elapsed from the onset of the first disease symptom was probably longer in the study by Nossent et al (5).

In contrast to damage at death, early damage in our study was higher in the ED group. This finding may be suggestive of a more severe disease course in the ED patients (30,31). In comparison with LD group, the mean age at diagnosis in the ED group was >10 years higher, suggestive of a potential contributing role of comorbidities related to higher age, many of which may overlap with organ damage attributed to inflammation in SLE or glucocorticoid or immunosuppressive treatment. Additional effort should be put into screening for comorbidities and signs of (early) organ damage in patients diagnosed at a later age.

To our knowledge, our study is the first to consistently use two cut-offs between early and late death in SLE patients.

The main limitation of our study is its retrospective nature based on medical records, which probably led to underreporting of clinically relevant events. Different laboratory methods were used over the patients’ disease course. We were not able to analyze medication doses, disease activity, and socioeconomic features. We excluded patients with an overlap between SLE and another systemic autoimmune disease due to the heterogeneity of their clinical features and the problem of their attribution to SLE. We also excluded patients not seen within three years before death (12). Still, this did not prevent us from dealing with the lack of data at time of death. Finally, we cannot state that the results are representative for entire Croatia.

In conclusion, our study did not confirm the bimodal pattern of mortality and found no difference between the frequency of causes of early and late death in SLE patients. The role of stroke as a cause of exclusively late death requires elucidation. Matching hospital data with vital statistics databases is required to fully assess the impact of SLE on mortality.
