# Supplementary material for: Disease characteristics and causes of early and late death in a group of Croatian patients with systemic lupus erythematosus deceased over a 10-year period
Source: Croat Med J. 2018 Feb;59(1):3–12. doi: 10.3325/cmj.2018.59.3 (PMC5833101; doi:10.3325/cmj.2018.59.3)
Supplement: Supplementary Material 1 [file CroatMedJ_59_s001.pdf]

Supplementary material 1. A. Frequency of the American College of Rheumatology (ACR) criteria at death (cumulatively) and comparison of the frequencies between early and late death

ACR criterion (at death)	All (N=90)	%	Early death (<5 years) (n=21)	Late death (≥5 years) (n=69)	p	Early death (<10 years) (n=43)	Late death (≥10 years) (n=47)	p
Malar rash	46	51	6	40	0,018	17	29	0,036
Discoid rash	22	24	9	13	0,025	14	8	0,087
Photosensitivity	34	38	6	28	0,320	14	20	0,329
Oral ulcers	11	12	4	7	0,275	10	1	0,002
Nonerosive arthritis	63	70	11	52	0,044	29	34	0,613
Serositis	33	37	7	26	0,717	13	20	0,226
a) Pleuritis	27	30	6	21	0,870	12	15	0,679
b) Pericarditis	17	19	4	3	1,000	6	11	0,253
Renal disorder	49	54	9	40	0,223	20	29	0,148
a) Persistent proteinuria	40	44	8	32	0,504	16	24	0,186
b) Cellular casts	40	44	8	32	0,504	18	22	0,637
Neurologic disorder	11	12	3	8	0,714	6	5	0,632
a) Seiures	8	9	1	7	0,673	3	5	0,716
b) Psychosis	4	4	2	2	0,231	3	1	0,345
Hematologic disorder	75	83	16	59	0,328	31	44	0,006
a) Hemolytic anemia	11	12	5	6	0,120	8	3	0,077
b) Leukopenia	44	49	10	34	0,894	20	24	0,666
c) Lymphopenia	58	64	14	44	0,808	26	32	0,451
d) Thrombocytopenia	26	29	7	19	0,608	12	14	0,844
Immunologic disorder	83	92	17	66	0,049	38	45	0,252
a) anti-dsDNA antibodies	71	79	12	59	0,012	32	39	0,320
b) anti-Smith antibodies	12	13	3	9	1,000	6	6	0,869
c) anticardiolipin antibodies	46	51	9	37	0,388	20	26	0,404
d) lupus anticoagulant	13	14	3	10	1,000	6	7	0,899
Antinuclear antibodies	86	96	20	66	1,000	41	45	1,000

P<0.002 considered statistically significant,
after adjustment for multiple comparisons

Supplementary material 1. B. Frequency of the American College of Rheumatology (ACR) criteria at diagnosis and comparison of the frequencies between early and late death

ACR criterion (at diagnosis)	All (N=73)	%	Early death (<5 years) (n=21)	Late death (≥5 years) (n=52)	p	Early death (<10 years) (n=43)	Late death (≥10 years) (n=30)	p
Malar rash	24	33	4	20	0,110	12	12	0,279
Discoid rash	16	22	9	7	0,011	12	4	0,139
Photosensitivity	16	22	5	11	0,765	9	7	0,807
Oral ulcers	5	7	3	2	0,140	5	0	0,074
Nonerosive arthritis	47	64	11	36	0,174	28	19	0,876
Serositis	15	21	7	8	0,112	10	5	0,493
a) Pleuritis	14	19	6	8	0,207	9	5	0,649
b) Pericarditis	6	8	4	2	0,053	5	1	0,390
Renal disorder	23	32	9	14	0,185	16	7	0,209
a) Persistent proteinuria	18	25	7	11	0,274	12	6	0,441
b) Cellular casts	17	23	6	11	0,547	12	5	0,264
Neurologic disorder	6	8	3	3	0,345	5	1	0,390
a) Seiures	3	4	1	2	1,000	2	1	1,000
b) Psychosis	3	4	2	1	0,197	3	0	0,264
Hematologic disorder	34	47	13	21	0,095	22	12	0,347
a) Hemolytic anemia	4	5	1	3	1,000	3	1	0,639
b) Leukopenia	22	30	7	15	0,705	14	8	0,589
c) Lymphopenia	20	27	10	10	0,014	17	3	0,005
d) Thrombocytopenia	9	12	4	5	0,269	7	2	0,292
Immunologic disorder	46	63	14	32	0,681	32	14	0,016
a) anti-dsDNA antibodies	38	52	10	28	0,630	28	10	0,008
b) anti-Smith antibodies	1	1	1	0	0,288	1	0	1,000
c) anticardiolipin antibodies	11	15	6	5	0,067	10	1	0,022
d) lupus anticoagulant	4	5	3	1	0,069	3	1	0,639
Antinuclear antibodies	63	86	19	44	0,714	40	23	0,080

P<0.002 considered statistically significant, after adjustment for multiple comparisons
