# Supplementary material for: Disease characteristics and causes of early and late death in a group of Croatian patients with systemic lupus erythematosus deceased over a 10-year period
Source: Croat Med J. 2018 Feb;59(1):3–12. doi: 10.3325/cmj.2018.59.3 (PMC5833101; doi:10.3325/cmj.2018.59.3)
Supplement: Supplementary Material 2 [file CroatMedJ_59_s002.pdf]

Supplementary material 2. A. Frequency of patients meeting each component of the Systemic Lupus International Collaborating Clinics/American College of Rheumatology Damage Index (SDI) at death, comparison of the frequencies between early and late death

Component of SDI at death	All (N=90)	%	Early death (<5 years) (n=21)	Late death (≥5 years) (n=69)	P	Early death (<10 years) (n=43)	Late death (≥10 years) (n=47)	P
Ocular	30	33	6	24	0.597	13	17	0.551
a) Cataract	28	31	5	23	0.409	12	26	0.530
b) Retinal change or optic atrophy	9	10	1	8	0.679	2	7	0.161
Neuropsychiatric	43	48	7	36	0.130	16	27	0.055
a) Cognitive impairment or major psychosis	17	19	2	15	0.340	6	11	0.253
b) Seizures	6	7	1	5	1,000	2	4	0.679
c) Cerebrovascular accident	21	23	4	17	0.771	7	14	0.130
d) Cranial or peripheral neuropathy	20	22	2	18	0.141	8	12	0.430
e) Transverse myelitis	0	0	0	0	NT	0	0	NT
Renal	25	28	3	22	0.115	11	14	0.656
a) Glomerular filtration rate <50%	21	23	2	19	0.139	10	11	0.987
b) Proteinuria >3.5 g/24h	7	8	1	6	1,000	2	5	0.438
c) End-stage renal disease	6	7	0	6	0.329	1	5	0.206
Pulmonary	20	22	4	16	0.774	6	14	0.071
a) Pulmonary hypertension	9	10	2	7	1,000	2	7	0.161
b) Pulmonary fibrosis	13	14	3	10	1,000	5	8	0.467
c) Shrinking lung	1	1	0	1	1,000	0	1	1,000
d) Pleural fibrosis	13	14	3	10	1,000	5	8	0.467
e) Pulmonary infarction	1	1	0	1	1,000	0	1	1,000
Cardiovascular	46	51	9	37	0.388	19	27	0.209
a) Angina or coronary artery bypass	15	17	2	13	0.506	5	10	0.220
b) Myocardial infarction	13	14	2	11	0.725	3	10	0.054
c) Cardiomyopathy	31	34	5	26	0.241	11	20	0.091

d) Valvular disease	15	17	4	11	0.744	8	7	0.637
e) Pericarditis for 6 months or pericardiectomy	1	1	0	1	1,000	0	1	1,000
Peripheral vascular	19	21	2	17	0.221	5	14	0.035
a) Claudication	8	9	0	8	0.190	3	5	0.716
b) Minor tissue loss	4	4	1	3	1,000	2	2	1,000
c) Significant tissue loss ever	8	9	0	8	0.19	1	7	0.060
d) Venous thrombosis	10	11	1	9	0.442	1	9	0.016
Gastrointestinal	7	8	0	7	0.193	1	6	0.113
a) Infarction or resection of bowel	4	4	0	4	0.569	0	4	0.118
b) Mesenteric insufficiency	0	0	0	0	NT	0	0	NT
c) Chronic peritonitis	0	0	0	0	NT	0	0	NT
d) Stricture or upper gastrointestinal tract surgery	3	3	0	3	1,000	1	2	1,000
Musculoskeletal	53	59	**5**	**48**	**<0.001**	18	35	0.002
a) Muscle atrophy or weakness	31	34	2	29	0.006	11	20	0.091
b) Deforming or erosive arthritis	15	17	1	14	0.177	6	9	0.509
c) Osteoporosis with fracture or vertebral collapse	27	30	2	25	0.019	8	19	0.024
d) Avascular necrosis	14	16	2	12	0.507	4	10	0.118
e) Osteomyelitis	1	1	0	1	1,000	0	1	1,000
Skin	15	17	3	12	1,000	5	10	0.220
a) Scarring chronic alopecia	7	8	0	7	0.193	2	5	0.438
b) Extensive scarring or panniculum other than scalp and pulp space	6	7	2	4	0.621	2	4	0.679
c) Skin ulceration	4	4	1	3	1,000	1	3	0.618
Premature gonadal failure (premature menopause)	6	9	0	6	0.325	1	5	0.197
Diabetes	15	17	3	12	1,000	7	8	0.925
Malignancy	22	24	1	21	0.017	5	17	0.007

P<0.001 considered statistically significant, after adjustment for multiple comparisons
bold with blue background - significant difference
NT - not tested

Supplementary material 2. B. Frequency of patients meeting each component of the Systemic Lupus International Collaborating Clinics/American College of Rheumatology Damage Index (SDI) one year following diagnosis, comparison of the frequencies between early and late death

Component of SDI one year after diagnosis	All (N=86)	%	Early death (<5 years) (n=17)	Late death (≥5 years) (n=69)	P	Early death (<10 years) (n=39)	Late death (≥10 years) (n=47)	P
Ocular	6	7	3	3	0.089	5	1	0.087
a) Cataract	4	5	2	2	0.174	3	1	0.325
b) Retinal change or optic atrophy	3	3	1	2	0.488	2	1	0.588
Neuropsychiatric	21	24	8	13	0.026	15	6	0.006
a) Cognitive impairment or major psychosis	7	8	2	5	0.621	6	1	0.043
b) Seizures	3	3	1	2	0.488	2	1	0.588
c) Cerebrovascular accident	9	10	4	5	0.071	6	3	0.289
d) Cranial or peripheral neuropathy	6	7	1	5	1,000	4	2	0.404
e) Transverse myelitis	0	0	0	0	NT	0	0	NT
Renal	4	5	2	2	0.174	4	0	0.039
a) Glomerular filtration rate <50%	3	3	1	2	0.488	3	0	0.089
b) Proteinuria >3.5 g/24h	1	1	1	0	0.198	1	0	0.454
c) End-stage renal disease	0	0	0	0	NT	0	0	NT
Pulmonary	4	5	2	2	0.174	3	1	0.325
a) Pulmonary hypertension	1	1	1	0	0.198	1	0	0.454
b) Pulmonary fibrosis	4	5	2	2	0.174	3	1	0.325
c) Shrinking lung	0	0	0	0	NT	0	0	NT
d) Pleural fibrosis	4	5	2	2	0.174	3	1	0.325
e) Pulmonary infarction	0	0	0	0	NT	0	0	NT
Cardiovascular	15	17	7	8	0.009	**13**	**2**	**<0.001**

a) Angina or coronary artery bypass	3	3	2	1	0.098	3	0	0.089
b) Myocardial infarction	3	3	2	1	0.098	3	0	0.089
c) Cardiomyopathy	7	8	2	5	0.621	6	1	0.043
d) Valvular disease	7	8	3	4	0.136	6	1	0.043
e) Pericarditis for 6 months or pericardiectomy	0	0	0	0	NT	0	0	NT
Peripheral vascular	4	5	1	4	1,000	1	3	0.623
a) Claudication	0	0	0	0	NT	0	0	NT
b) Minor tissue loss	0	0	0	0	NT	0	0	NT
c) Significant tissue loss ever	0	0	0	0	NT	0	0	NT
d) Venous thrombosis	4	5	1	4	1,000	1	3	0.623
Gastrointestinal	1	1	0	1	1,000	1	0	0.454
a) Infarction or resection of bowel	0	0	0	0	NT	0	0	NT
b) Mesenteric insufficiency	0	0	0	0	NT	0	0	NT
c) Chronic peritonitis	0	0	0	0	NT	0	0	NT
d) Stricture or upper gastrointestinal tract surgery	1	1	0	1	1,000	1	0	0.454
Musculoskeletal	15	17	3	12	1,000	10	5	0.068
a) Muscle atrophy or weakness	6	7	1	5	1,000	5	1	0.087
b) Deforming or erosive arthritis	3	3	0	3	1,000	2	1	0.059
c) Osteoporosis with fracture or vertebral collapse	6	7	1	5	1,000	4	2	0.404
d) Avascular necrosis	2	2	1	1	0.358	1	1	1,000
e) Osteomyelitis	0	0	0	0	NT	0	0	NT
Skin	7	8	2	5	0.621	4	3	0.697
a) Scarring chronic alopecia	3	3	0	3	1,000	2	1	0.059
b) Extensive scarring or panniculum other than scalp and pulp space	3	3	2	1	0.098	2	1	0.588
c) Skin ulceration	1	1	0	1	1,000	0	1	1,000
Premature gonadal failure (premature menopause)	2	3	0	2	1,000	1	1	1,000
Diabetes	8	9	3	5	0.190	6	2	0.133

Malignancy	2	2	1	1	0.358	2	0	0.203

P<0.001 considered statistically significant, after adjustment for multiple comparisons
bold with blue background - significant difference
NT - not tested
