# Supplementary material for: Disease characteristics and causes of early and late death in a group of Croatian patients with systemic lupus erythematosus deceased over a 10-year period
Source: Croat Med J. 2018 Feb;59(1):3–12. doi: 10.3325/cmj.2018.59.3 (PMC5833101; doi:10.3325/cmj.2018.59.3)
Supplement: Supplementary Material 3 [file CroatMedJ_59_s003.pdf]

Supplementary material 3. Time from diagnosis of systemic lupus erythematosus (SLE) to meeting each component of the Systemic Lupus International Collaborating Clinics/American College of Rheumatology Damage Index (SDI) (median, minimum and maximum for each item)

Component of SDI	Deceased (N=90)	%	Median (years)	Minimum (years)	Maximum (years)
Ocular	30	33	4,5	0,5	28
a) Cataract	28	31	4,5	0,5	29
b) Retinal change or optic atrophy	9	10	11	0,5	28
Neuropsychiatric	43	48	2	0,5	28
a) Cognitive impairment or major psychosis	17	19	4	0,5	32
b) Seizures	6	7	4,75	0,5	16
c) Cerebrovascular accident	25	28	8	0,5	33
d) Cranial or peripheral neuropathy	20	22	4	0,5	28
e) Transverse myelitis	0	0	NA	NA	NA
Renal	25	28	6	0,5	20
a) Glomerular filtration rate <50%	21	23	6	0,5	32
b) Proteinuria >3.5 g/24h	7	8	6	0,5	15
c) End-stage renal disease	6	7	8,5	2	28
Pulmonary	20	22	10	0,5	30
a) Pulmonary hypertension	9	10	10	0,5	33
b) Pulmonary fibrosis	13	14	3	0,5	30
c) Shrinking lung	1	1	23	23	23
d) Pleural fibrosis	13	14	3	0,5	30
e) Pulmonary infarction	1	1	10	10	10
Cardiovascular	46	51	4,5	0,5	33
a) Angina or coronary artery bypass	15	17	5	0,5	27
b) Myocardial infarction	18	20	7	0,5	34
c) Cardiomyopathy	31	34	9	0,5	33
d) Valvular disease	15	17	2	0,5	27
e) Pericarditis for 6 months or pericardiectomy	1	1	11	11	11
Peripheral vascular	19	21	5,5	0,5	23

a) Claudication	8	9	6	3	23
b) Minor tissue loss	4	4	7,5	0,5	17
c) Significant tissue loss ever	8	9	11	7	34
d) Venous thrombosis	10	11	3,5	0,5	21
Gastrointestinal	7	8	6	0,5	10
a) Infarction or resection of bowel	4	4	6	3	10
b) Mesenteric insufficiency	0	0	NA	NA	NA
c) Chronic peritonitis	0	0	NA	NA	NA
d) Stricture or upper gastrointestinal tract surgery	3	3	2	0,5	8
Musculoskeletal	53	59	5	0,5	33
a) Muscle atrophy or weakness	31	34	5	0,5	25
b) Deforming or erosive arthritis	15	17	6	0,5	16
c) Osteoporosis with fracture or vertebral collapse	27	30	5	0,5	33
d) Avascular necrosis	19	21	5,5	0,5	17
e) Osteomyelitis	1	1	8	8	8
Skin	15	17	2	0,5	14
a) Scarring chronic alopecia	7	8	2	0,5	14
b) Extensive scarring or panniculum other than scalp and pulp space	6	7	1,75	0,5	12
c) Skin ulceration	4	4	5,5	1	12
Premature gonadal failure (premature menopause)	6	9	5	0,5	10
Diabetes	15	17	1	0,5	30
Malignancy	25	28	10	0,5	30

NA - not applicable
